# Factors affecting peak impact force during soccer headers and implications for the mitigation of head injuries

**DOI:** 10.1371/journal.pone.0240162

**Published:** 2020-10-16

**Authors:** Joshua Auger, Justin Markel, Dimitri D. Pecoski, Nicolas Leiva-Molano, Thomas M. Talavage, Larry Leverenz, Francis Shen, Eric A. Nauman

**Affiliations:** 1 School of Mechanical Engineering, Purdue University, West Lafayette, Indiana, United States of America; 2 Weldon School of Biomedical Engineering, Purdue University, West Lafayette, Indiana, United States of America; 3 School of Electrical and Computer Engineering, Purdue University, West Lafayette, Indiana, United States of America; 4 Department of Health and Kinesiology, Purdue University, West Lafayette, Indiana, United States of America; 5 University of Minnesota Law School, University of Minnesota, Minneapolis, Minnesota, United States of America; 6 Department of Basic Medical Sciences, Purdue University, West Lafayette, Indiana, United States of America; Universitat de Valencia, SPAIN

## Abstract

It has been documented that up to 22% of all soccer injuries are concussions. This is in part due to players purposely using their head to direct the ball during play. To provide a more complete understanding of head trauma in soccer athletes, this study characterized the effects of four soccer ball characteristics (size, inflation pressure, mass, velocity) on the resulting peak impact force as it relates to the potential for incurring neurophysiological changes. A total of six hundred trials were performed on size 4 and 5 soccer balls as well as a novel lightweight soccer ball. Impact force was measured with a force plate and ball velocity was determined using motion capture. These data were used, in conjunction with dimensional analysis to relate impact force to ball size, mass, velocity, and pressure. Reasonable reductions in allowable ball parameters resulted in a 19.7% decrease in peak impact force. Adjustments to ball parameters could reduce a high cumulative peak translational acceleration soccer athlete down into a previously defined safer low loading range. In addition, it was noted that water absorption by soccer balls can result in masses that substantially increase impact force and quickly surpass the NCAA weight limit for game play. Additional research is required to determine whether varying soccer ball characteristics will enable soccer players to avoid persistent neurophysiological deficits or what additional interventions may be necessary and the legal implications of these data are discussed.

## 1 Introduction

Soccer is the most popular sport in the world, with more than 265 million professional and amateur level players worldwide, and it has been documented that up to 22% of all the injuries in the sport are concussions [1–4]. This is, in part, due to players purposely using their head to direct the ball during play [3]. A soccer player will commonly experience up to 12 headers over the course of a single game [5]. Across a single season, a professional soccer player performs an average of 800 headers, excluding those performed during practice. Several studies have already shown that concussion rates in soccer are comparable to, and in some cases exceed, those in football and ice hockey [2, 5–9]. Moreover, previous studies have demonstrated that more than 70% of concussed soccer players did not realize they had suffered a concussion during the season [6, 10].

It must be noted that an official diagnosis of concussion is not required for athletes to exhibit substantial changes in brain structure and function that are detectable with a range of assessment tools [11–17]. Recent work suggests that some aspect of cumulative exposure is the primary risk factor in the development of pathological neurophysiological changes accrued throughout the season in contact sports [11, 16, 18–20] and that impacts exceeding 50g may be particularly dangerous [21].

Previous studies have demonstrated that ball velocity and the mass ratio between the ball and the player are important parameters when categorizing potential injury risk [22–24]. According to the 2016-2017 NCAA Men’s and Women’s Soccer Rule Book, Rule 2, Subsection 2.1, the soccer ball must have a circumference of 68.6-71.1 cm, a pressure range of 0.61-1.12 bar (8.8-16.2 psi), and a weight between 396.9-453.6 g at the start of game play and may weigh up to 474.9 g as a result of water absorption and use [25]. Similarly, the 2015-2016 FIFA Laws Of The Game, Law 2, allows for a soccer ball with a circumference of 68-70 cm, a pressure range of 0.59-1.08 bar (8.5-15.6 psi), and a weight between 410-450 g at the start of game play with no upper limit on ball weight as a result of water absorption and use [26]. Rules and regulations can vary with age group. For instance, in the United States, size 4 soccer balls are typically required under 12 years of age [27].

The goal of this study was to characterize the effects of soccer ball parameters (size, inflation pressure, mass, velocity) on the resulting peak impact force and correlate it to the potential for incurring injury. A dimensional analysis served to develop a predictive equation involving the examined parameters. A sensitivity analysis was then used to ascertain the contribution of these parameters to the peak impact force and isolate the most important factors. From these findings, recommendations will be made for further work that may lead to improvements in safety and reduced injury-risk for soccer players.

## 2 Theory

### 2.1 Dimensional analysis

A dimensional analysis was conducted with peak impact force as the output and the four most important inputs were assumed to be inflation pressure (*p*), mass (*m*), diameter (*d*), and incoming velocity (*v*). Assuming we have captured all the relevant parameters, we can assert that,

$$\begin{array}{c} F_{p}=f\left(p,m,d,v\right). \end{array}$$ (1)

For Eq (1), there were five physical variables expressed in terms of three independent physical units of mass, length, and time, denoted by M, L, and T, respectively. The Buckingham Pi Theorem states that Eq (1) can be rescaled into an equivalent dimensionless relationship having only two dimensionless parameters [28]. For this study, *m*, *d*, *v* were selected to provide independent physical units, and subsequently the dimensionless output parameter, Π_*o*_, was expressed as,

$$\begin{array}{c} \Pi_{o}=\frac{F_{p}d}{mv^{2}}, \end{array}$$ (2)

and the input parameter, Π_*i*_, can be written as,

$$\begin{array}{c} \Pi_{i}=\frac{pd^{3}}{mv^{2}}. \end{array}$$ (3)

Thus Eq (1) can be reformulated as,

$$\begin{array}{c} \Pi_{o}=f\left(\Pi_{i}\right). \end{array}$$ (4)

This relation is assumed to follow intermediate asymptotic behavior and therefore takes the form, $f\left(\Pi_{i}\right)=B\Pi_{i}^{\beta }$ [28]. If all relevant parameters influencing the peak impact force have been included, then the natural logarithm of the Π variables yields,

$$\begin{array}{c} \text{ln}\left(\Pi_{o}\right)=\text{ln}\left(B\right)+\beta \text{ln}\left(\Pi_{i}\right), \end{array}$$ (5)

which allows us to determine the coefficients *B* and *β* from a linear regression of the experimental data. Once these coefficients are determined, the equation for the peak impact force is given by,

$$\begin{array}{c} F_{p}=(\frac{Bmv^{2}}{d}){\left(\frac{pd^{3}}{mv^{2}}\right)}^{\beta }. \end{array}$$ (6)

## 3 Methods

### 3.1 Experimental parameters

Data were collected for six soccer balls; two size 4, two size 5, and two novel “lightweight” balls donated by EIR Soccer (hereafter referred to as size 4.5 per manufacturer labeling on the ball). The size 4 and size 5 were both Adidas Starlancer^™^ soccer balls with the same typical hexagon/pentagon tessellating panel construction. The manufacturer-specified pressure ranges are printed on every ball and for the size 4, 4.5, and 5 balls they were 0.50-0.70 bar (7.3-10.2 psi), 0.60-0.80 bar (8.7-11.6 psi), and 0.50-0.70 bar (7.3-10.2 psi), respectively. It is worth noting that manufacturer pressure specifications are much narrower than and on the lower end of game play regulations. Each size soccer ball underwent testing at four different pressures: 0.27, 0.55, 0.83, and 1.10 bar (4, 8, 12, and 16 psi). Testing at 0.27 bar (4 psi) was included to examine each of the soccer balls when pressurized below the standard manufacturing pressure specifications. For each soccer ball undergoing testing, the size and mass at each pressure were measured.

### 3.2 Kicking procedure and data collection

**Fig 1:**
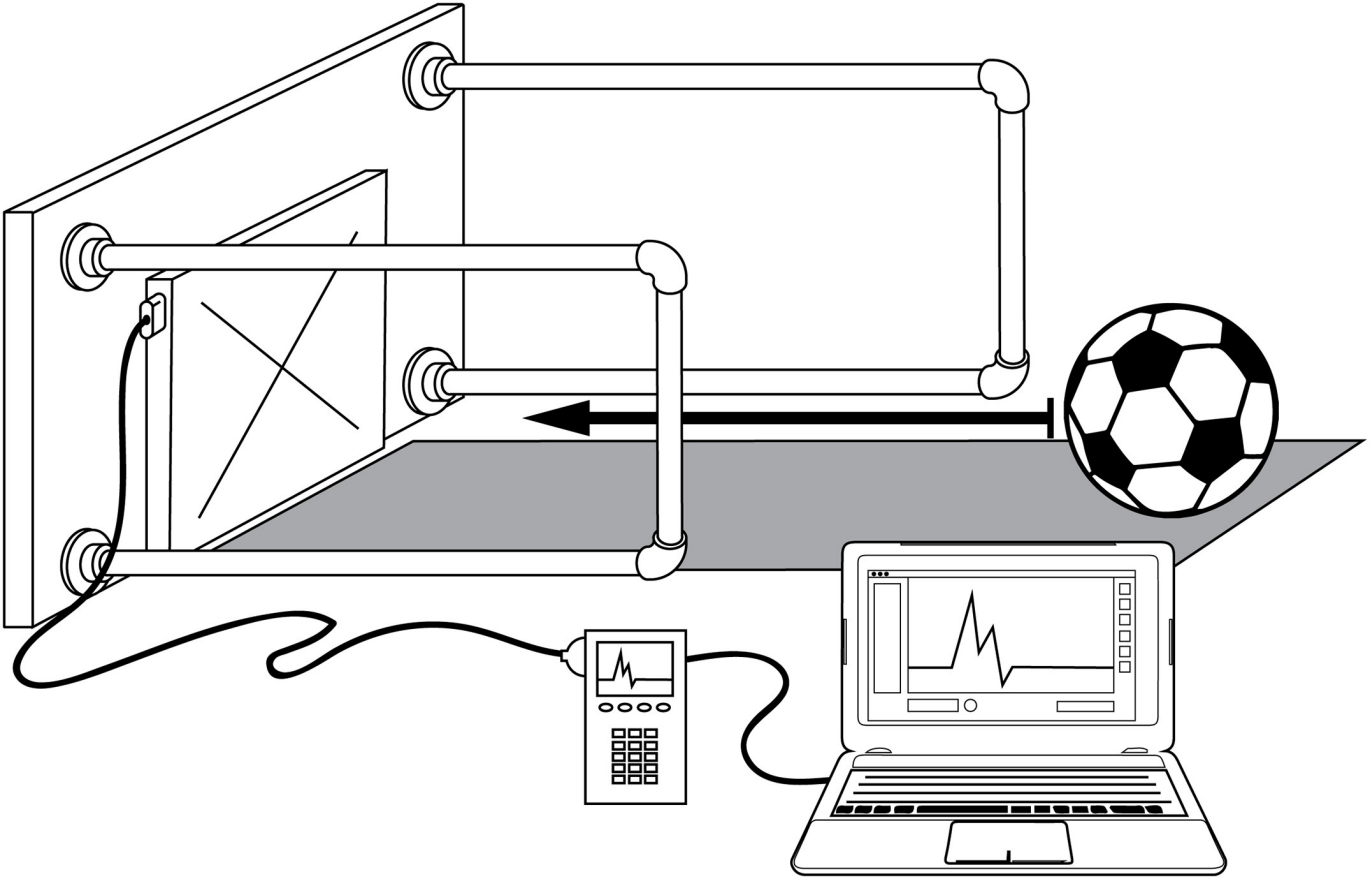
Experimental set-up used for each kick of a soccer ball. The vertically mounted force plate collected impact force data through the PASCO Xplorer GLX^™^ sensor receiver unit connected to a laptop running PASCO Capstone Software^™^ [29]. A camera mounted on a crossbar above the force plate captured impact motion for calculating ball velocity.

A motion capture system with an integrated force plate ws used to obtain data for this experiment (Fig 1). Each soccer ball was kicked by a researcher using a typical instep strike in sets of ten, ranging from low to high power kicks within each set, from a distance of approximately 2 meters away from the force plate. Non-standard kicks resulting in ricochet or irregular angled trajectories were retried until a clean, perpendicular trajectory was achieved. After each set of ten kicks, the soccer ball pressure was checked using a pressure gauge and adjusted as necessary to maintain the correct pressure level for the test. This procedure was repeated for 50 trials of each ball size at each pressure; 200 kicks per ball size, resulting in 600 total kicks.

### 3.3 Impact force measurement

Impact force data were collected using a PASCO Force Plate^™^ (PASCO, Roseville, CA, USA) mounted vertically on a metal back plate with brace beams on either side (Fig 1). The rig provided a rigid structure upon which the force plate was mounted to reduce any measurement noise from dampening vibration motion.

The PASCO Force Plate^™^ output the normal impact force and, when coupled with the PASCO Xplorer GLX^™^ sensor receiver unit, was capable of recording at a rate of 2 kHz [29]. Prior to each set of testing, the force plate was connected and tared to zero output to eliminate any resting noise. The force plate and sensor receiver were connected directly to a computer running the PASCO Capstone Software^™^ for data collection. A positive trigger was set at 4 N, which prompted a 40.0 ms data collection window (5.0 ms post-trigger and a 35.0 ms pre-trigger) to ensure the entire response wave of each impact was measured. A custom MATLAB script was then used to identify and index the peak impact force for each impact waveform [30].

### 3.4 Velocity measurement

A top-mounted video camera collected footage at 240 frames-per-second as the soccer ball approached the force plate, the deformation against the force plate, and the exit trajectory of the soccer ball (Fig 1). A stationary measuring stick on the contrasted background was used as a reference for distance in each video clip. For each trial, the video footage was separated into individual frames so the travel distance and velocity for the incoming and exiting trajectories could be determined.

### 3.5 Water submersion testing

A submersion experiment was conducted to examine the potential for mass increase during a 90-minute regulation game duration. According to the 2016-2017 NCAA Soccer Rule Book, the regulation soccer ball “shall not be more than 16 ounces nor less than 14 ounces” at the start of the game, and cannot “exceed 16.75 ounces even when wet and used” [25]. Converted to grams, the soccer ball must weight between 396.9-453.6 grams at the start of the game, and cannot exceed 474.9 grams from water absorption or use during game play. The 2015-2016 FIFA Laws Of The Game make no statements regarding limits to weight increase from water absorption. All three sizes of soccer balls were inflated to 0.55 bar (8-psi) to be within manufacturer specification as well as the lower limit of NCAA and FIFA specification. The soccer balls were weighed on a scale at the start of the experiment and then submerged up to the centerline of the ball. At 15 minute intervals over a 90 minute period, the three soccer balls were weighed and rotated.

### 3.6 Sensitivity analysis

Cotter’s method was used to perform a sensitivity analysis of the contribution of the aforementioned input parameters in the predictive equation from the dimensional analysis to isolate the most important parameters affecting the peak impact force [31, 32]. The range of values for each parameter were based on allowable professional-level (size 5) soccer game play regulations and values obtained from the literature (Table 1). These data provided the high and low values used for Cotter’s method. The minimum velocity corresponds to the in-field measured throw-in velocities and the maximum velocity corresponds to in-field measured shots-on-goal.

**Table 1:** Parameter ranges for sensitivity analysis.

Variable (units)	Minimum	Maximum	Source
Ball Mass (kg)	0.397	0.480	[25, 26]
Ball Diameter (m)	0.204	0.221	[25, 26]
Inflation Pressure (Pa)	58600	111700	[25, 26]
Velocity (m/s)	15	30	[22, 33]

In order to determine the effects of playing in the rain, a second sensitivity analysis was conducted to examine those changes that may occur when the broader mass range due to water absorption was taken into account. The direct submersion procedure of the experiment can be regarded as an extreme case of mass increase due to water absorption, representing the maximum possible mass increase.

## 4 Results

### 4.1 Physical characteristics of soccer balls

The size and mass for each ball at each pressure were measured three times and the average recorded (Table 2). While there was an 11% increase in mass when transitioning from the size 4 ball to the size 5 ball, the mass of the size 4.5 ball was consistently less than that of the size 4 ball.

**Table 2:** Dry soccer ball metrics by size.

Pressure (bar)	Mass (kg)	Diameter (m)
	Size 4 Ball	
0.27	0.387	0.204
0.55	0.388	0.205
0.83	0.389	0.206
1.10	0.391	0.206
	Size 4.5 Ball	
0.27	0.379	0.213
0.55	0.381	0.214
0.83	0.382	0.215
1.10	0.384	0.215
	Size 5 Ball	
0.27	0.430	0.211
0.55	0.431	0.220
0.83	0.433	0.221
1.10	0.435	0.221

### 4.2 Impact forces

**Fig 2:**
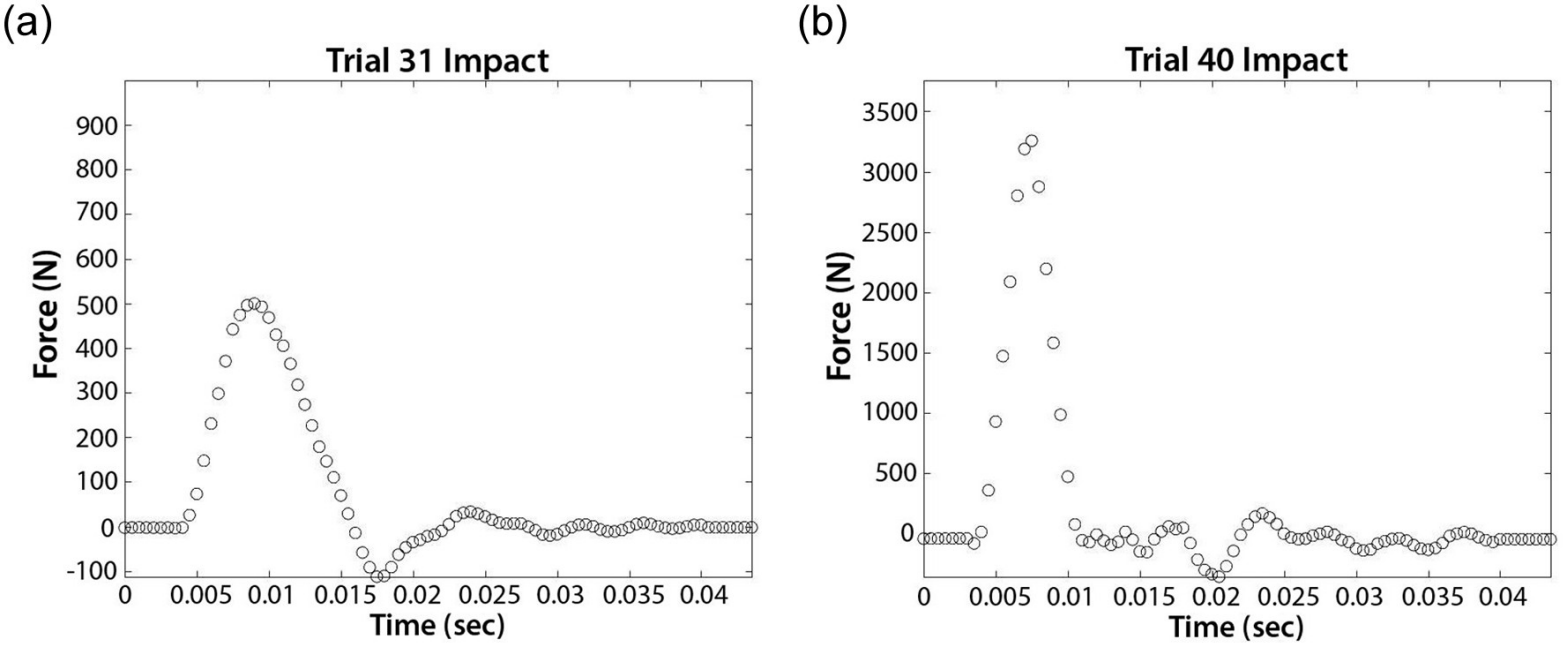
Two sample impact force waves. The first was taken from a size 4 soccer ball inflated to a pressure of 27.6 kPa and delivered at a speed of 4.57 m/s. The second was taken from a size 5 ball inflated to a pressure of 34.5 kPa and delivered at a speed of 14.6 m/s.

Force time series were collected from the force plate for every impact, providing the peak impact force, duration of impact, and impulse. The data ranges of peak impact force, and impact duration for the size 4 ball (across all tested pressures) were 201-4419 N, and 0.0055-0.017 s, respectively; for the size 4.5 ball: 355-4641 N, and 0.0045-0.017 s, respectively; and for the size 5 ball: 196-4667 N, and 0.0055-0.017 s, respectively (Fig 2).

Higher velocity kicks resulted in higher peak impact force at all pressures. Size 4 and size 4.5 soccer balls exhibited the same trends between velocity and impact force for increased pressure; increased soccer ball inflation pressure corresponded to increased peak impact force across the entire velocity range. The average peak impact force of all trials within the chosen velocity range demonstrated that decreased ball inflation pressure across all sizes resulted in decreased peak impact force (Table 3). Of particular note, the lightweight size 4.5 ball exhibited lower average peak impact forces across the entire pressure range. A diameter between a standard size 4 and size 5 soccer ball, while having a lower weight than both standard sizes, may have contributed to this observed difference. Considering a professional regulation size 5 soccer ball, a ball inflation pressure of 1.10 bar (16 psi) yields an average peak impact force of 3606 N. A ball inflation pressure of 0.55 bar (8 psi) yields an average peak impact force of 2895 N; a 20% decrease in average peak impact force for the same 14-17 m/s velocity range.

**Table 3:** Mean peak impact force (N) (+/- Standard Deviation) of all experimental trials within a 14-17 m/s velocity range.

Pressure	Size 4	Size 4.5	Size 5
0.28 bar (4 psi)	2508 (± 368)	2440 (± 340)	2669 (± 136)
0.55 bar (8 psi)	2858 (± 455)	2688 (± 380)	2895 (± 817)
0.83 bar (12 psi)	3167 (± 444)	2961 (± 346)	3284 (± 60)
1.10 bar (16 psi)	3644 (± 334)	3093 (± 326)	3606 (± 340)

### 4.3 Water submersion testing

Initial observations demonstrated that the majority of water absorption occurred in the first 15 minutes of submersion, with the size 4, 4.5, and 5 soccer balls increasing in mass by 16.3%, 5.7%, and 22.3%, respectively (Table 4).

**Table 4:** Change in soccer ball mass (kg) during water submersion.

t (min)	Size 4	Size 4.5	Size 5
0	0.389	0.380	0.431
15	0.465	0.403	0.555
30	0.462	0.417	0.586
45	0.462	0.418	0.584
60	0.475	0.418	0.585
75	0.471	0.419	0.592
90	0.482	0.421	0.582

### 4.4 Dimensional analysis

**Fig 3:**
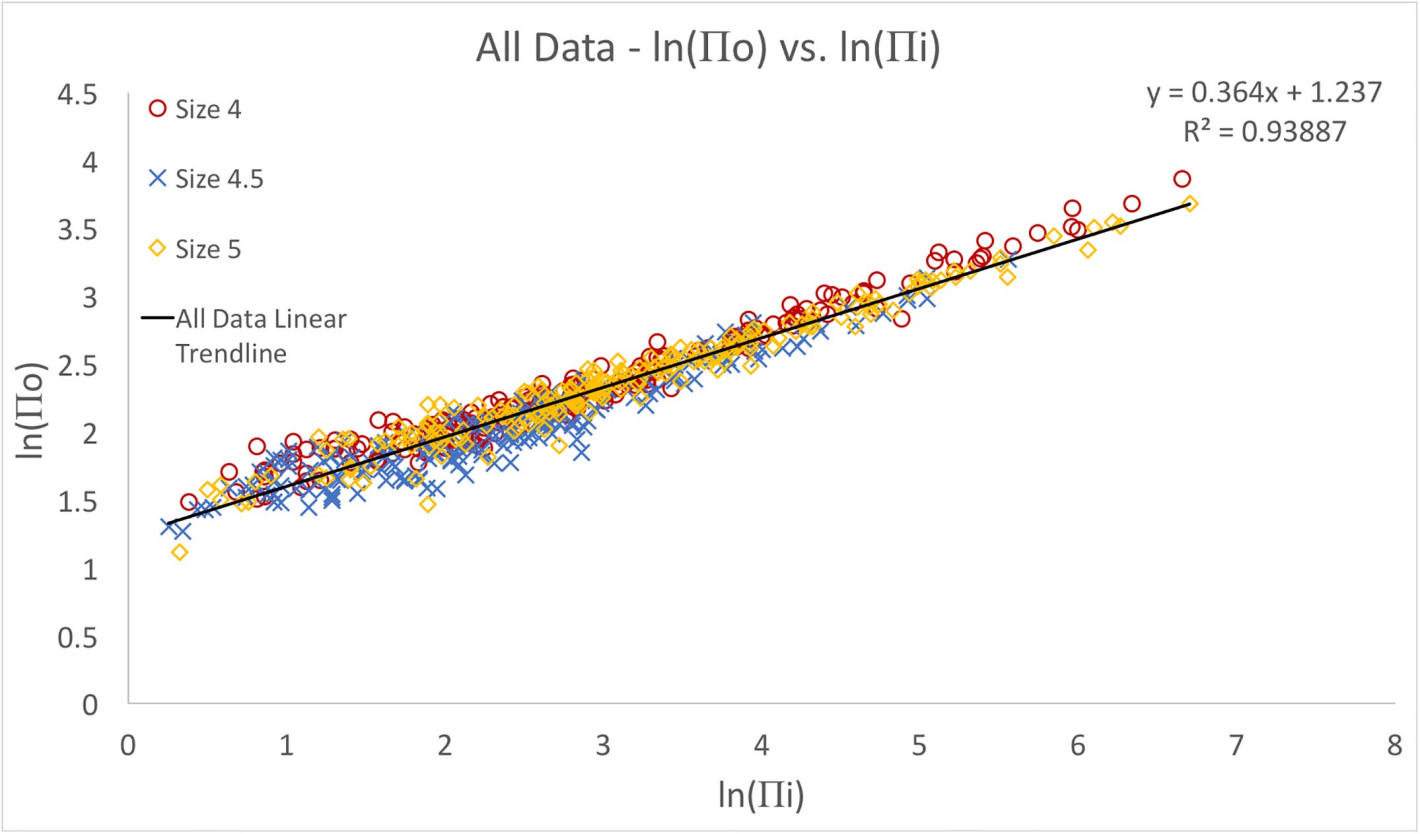
Natural log of Π_*o*_ (Force) versus Π_*i*_ of all 600 data points. A distinct linear relationship suggests all necessary parameters have been considered and all soccer balls follow the same theoretical peak force relationship.

The linear relationship between the the log transformed dimensionless variables was demonstrated across all ball sizes and inflation pressures (Fig 3) using Eqs 2 and 3 (*R*^2^ = 0.94). The corresponding equation for peak force was found to be,

$$\begin{array}{c} F_{p}=3.445(\frac{mv^{2}}{d}){\left(\frac{pd^{3}}{mv^{2}}\right)}^{0.364}. \end{array}$$ (7)

### 4.5 Sensitivity analysis

**Fig 4:**
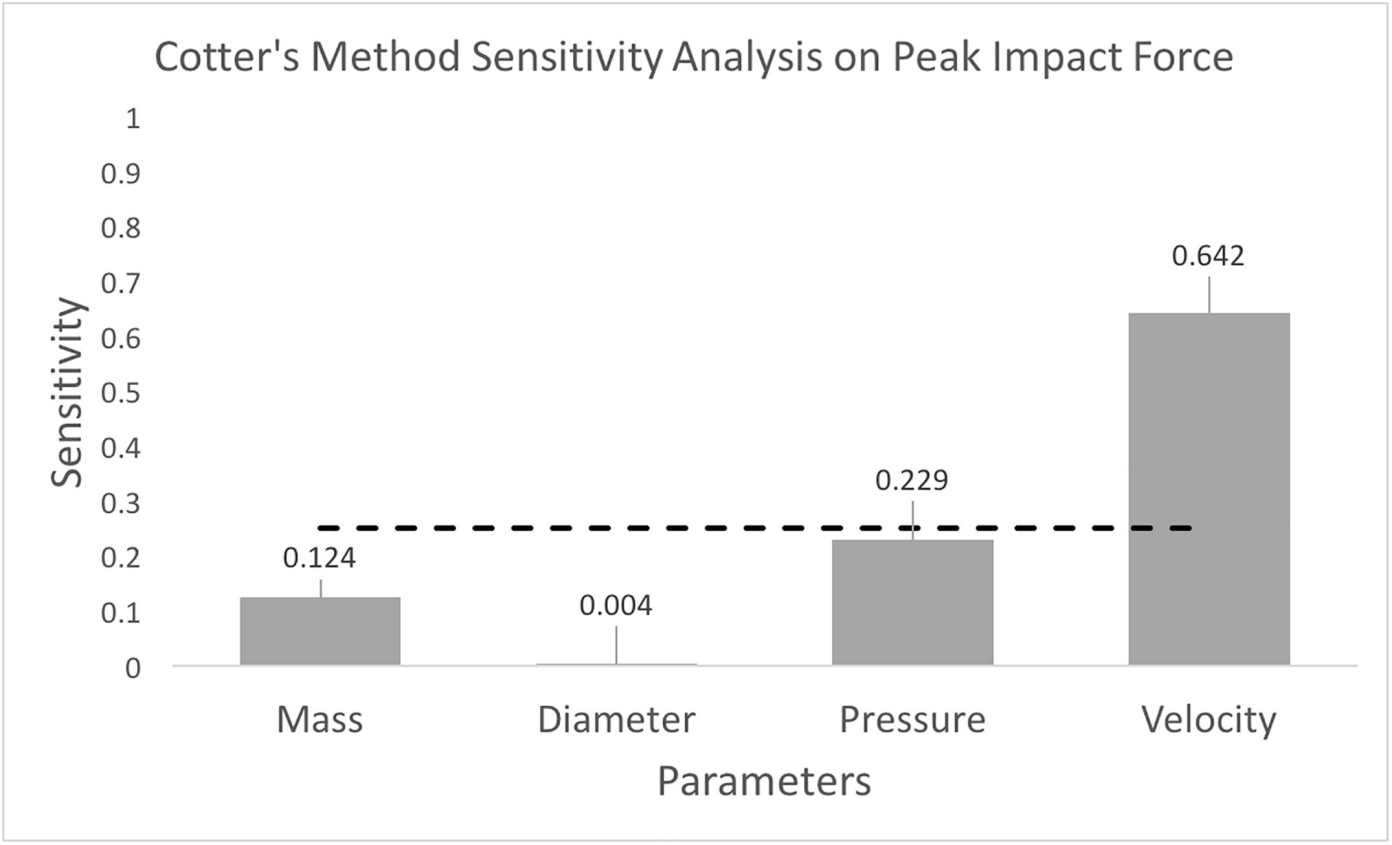
Cotter’s Method sensitivity analysis considering a mass range of 0.3969—0.4797 kg, diameter range of 0.2041–0.2208 m, inflation pressures between 58.6 and 111.7 kPa, and a velocity range of 15-30 m/s. The most important parameter was the ball velocity, but it should be noted that ball pressure exhibited a sensitivity value near the critical threshold of 0.25.

**Fig 5:**
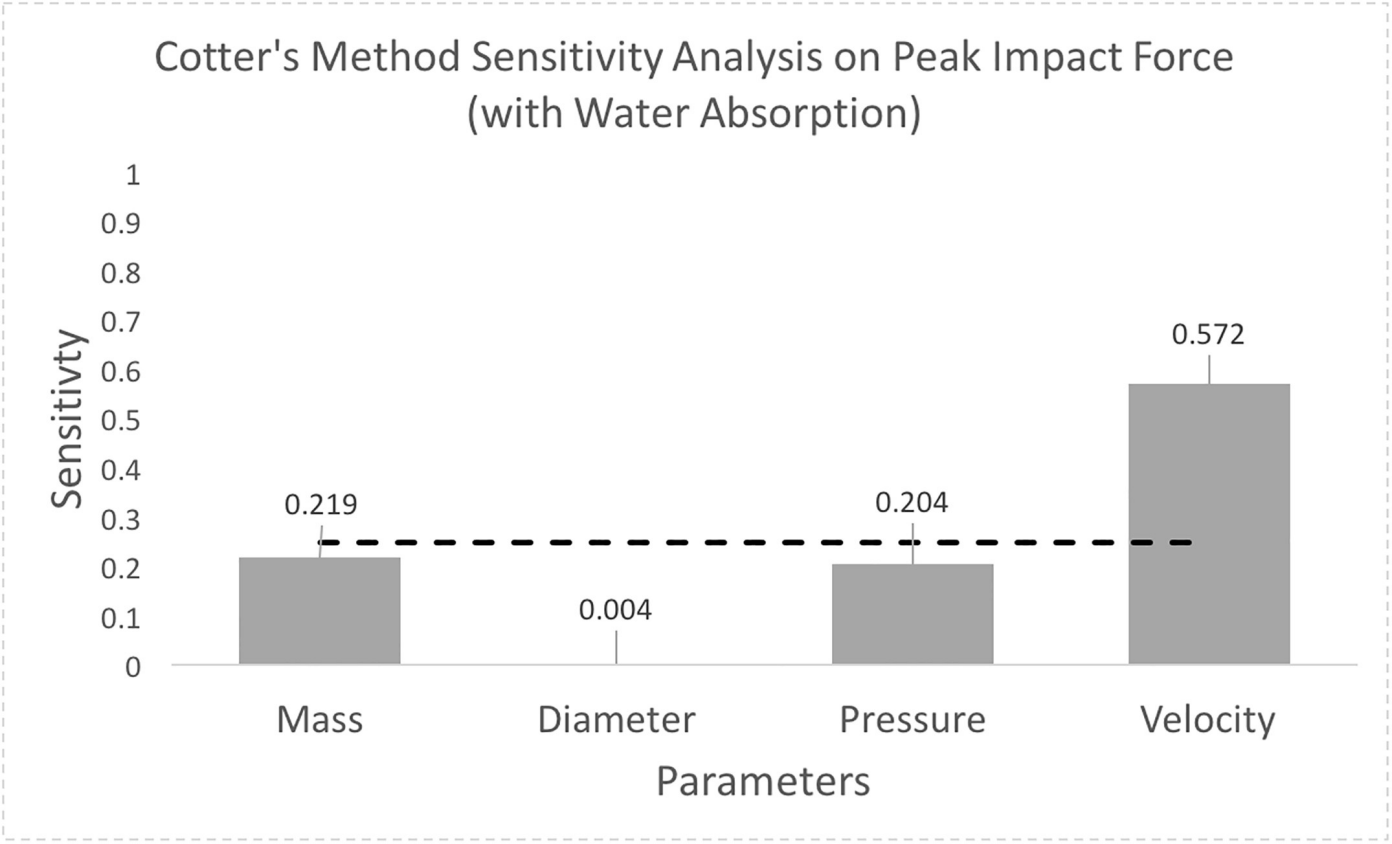
Cotter’s Method sensitivity analysis taking into account the increased water absorption mass value range for a professional-level size 5 soccer ball. Water absorption makes mass the second most important parameter.

The sensitivity analysis in both cases (without and with water absorption mass ranges) indicated that the most important factor affecting peak impact force was the ball velocity, with inflation pressure near the sensitivity threshold of 1/*n*_*p*_ = 0.25. (Figs 4 and 5). For mass, the sensitivity index was below 1/*n*_*p*_ at S = 0.12, when not accounting for water absorption (Fig 4). When accounting for the increased mass range due to water absorption, the sensitivity index increases to S = 0.219 and surpasses the sensitivity index of inflation pressure, S = 0.204 (Fig 5).

## 5 Discussion

Safety equipment exists for soccer, but it has not been widely adopted nor has such equipment been subjected to proper clinical validation as a preventative measure for decreasing risk of TBI from repetitive head impacts. Certain protective headband products have exhibited marginal force attenuation, but have yet to demonstrate a proven decrease in neurological impairment from head acceleration events [24, 34, 35]. Critically, the design of protective equipment and better understanding of the mechanisms by which repeated head trauma induces neurologic changes requires a thorough characterization of the forces generated during head contact. Therefore, the goal of this study was to characterize the effects of soccer ball parameters (size, inflation pressure, mass) across a velocity spread on the resulting peak impact force and correlation to translational acceleration as it relates to the potential for incurring injury.

The sensitivity analysis of the resultant equation for peak impact force showed that, as expected, the impact velocity of the soccer ball is the most influential factor (Fig 4). However, ball velocity is not necessarily controllable during game play and limiting ball velocity in game play would be impractical. Examining reasonably controllable parameters, the lowering of inflation pressure provides the greatest “rate of return” on decreasing peak impact force (Table 3). For typical ball velocities, a decrease in pressure from 1.10 bar (16 psi) to 0.55 bar (8 psi) drops the peak impact force by nearly 20%.

Water submersion testing demonstrated that soccer balls quickly absorb water and increase in mass (Table 4). Within 15 minutes of submersion, the size 5 soccer ball had already surpassed the upper 474.8 g weight limit allowable by NCAA rules [25]. Considering these values, a sensitivity analysis accounting for a larger mass range due to water absorption showed the sensitivity index for mass surpasses that of inflation pressure and sits right at the 1/*n*_*p*_ sensitivity threshold (Fig 5). The mass range assessed represents an ideal extreme water exposure case and actual environmental conditions with consistent game play may limit the level of water retention. However, these results make it evident that soccer ball mass does increase during water exposure and, if not consistently checked, can lead to mass increases beyond allowed limits.

Quantifying the effect that ball velocity, pressure, mass, and diameter have on impact force makes it possible to estimate the potential reduction in brain trauma that may be achieved by simple interventions. Archetypal biomechanical models demonstrate a nearly linear relation between peak impact force and peak translational acceleration of the center of mass of the head, meaning any decrease in impact force should provide a similar or greater perceived decrease in head acceleration.

**Fig 6:**
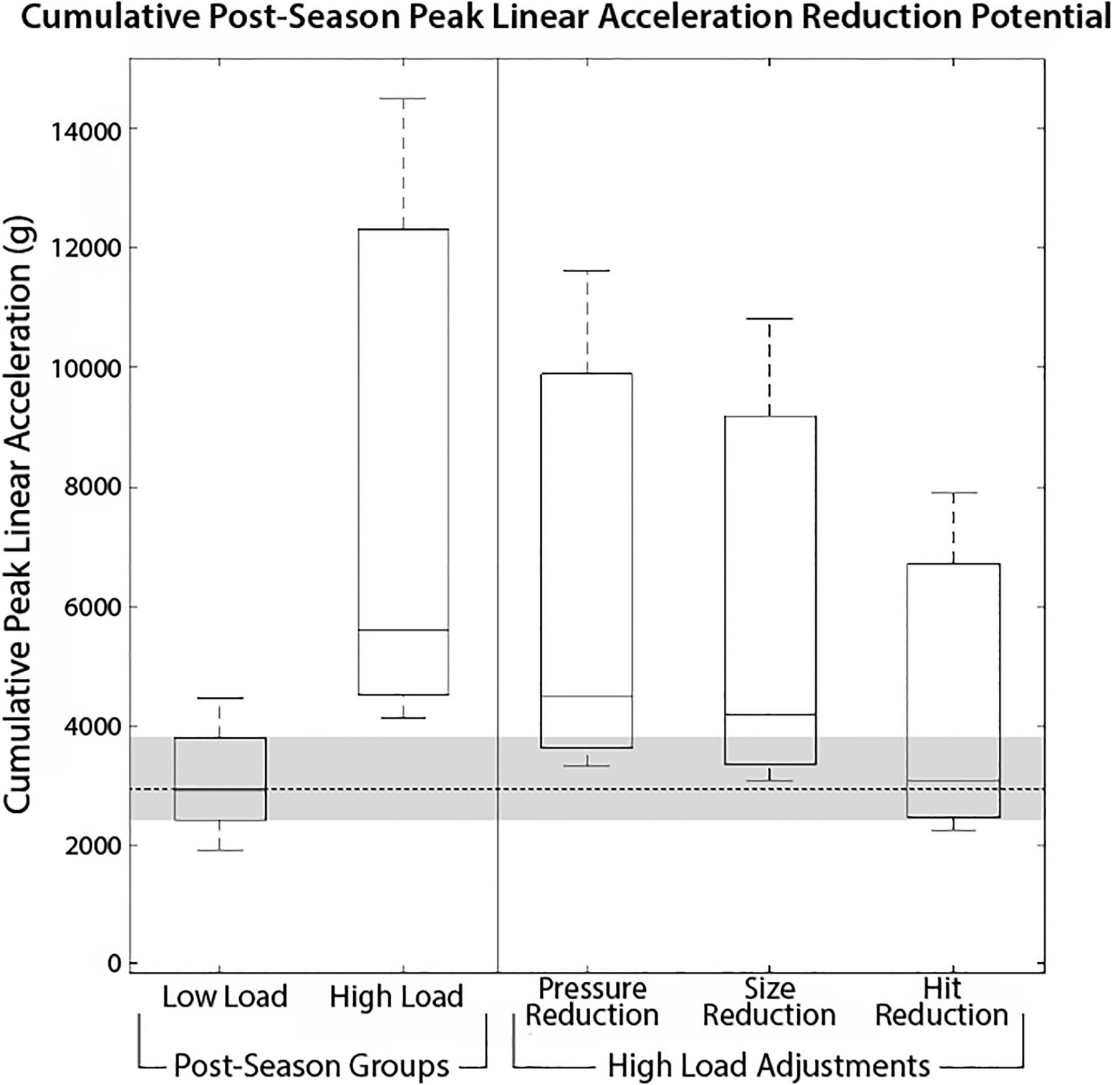
Svaldi et al.’s “High Load” and “Low Load” groups from a soccer season (High Load percentiles: 25th = 4515 g, 50th = 5615 g, 75th = 12313 g; Low Load percentiles: 25th = 2425 g, 50th = 2930 g, 75th = 3800 g) [36]. Post-season adjustments to the High Load group: 19.7% reduction from decreasing inflation pressure from 1.10 bar (16 psi) to 0.55 bar (8 psi), 7.1% reduction from downsizing a size 5, 0.55 bar (8 psi) ball to size 4.5, 0.55 bar (8 psi) ball (Table 3), and a 20% reduction in number of hits based on average PLA per hit (Adjusted High Load percentiles: 25th = 2463 g, 50th = 3063 g, 75th = 6718 g).

A 2016 study by Svaldi et al. paired functional MRI with head impact monitoring to track cerebrovascular reactivity (CVR) changes throughout a season of high school soccer [36]. Soccer athletes from two schools (n = 14; ages 15-17) were characterized as “High Load” or “Low Load”, according to cumulative PLA (High Load cumulative PLA group: 25th percentile = 4514.7 g, 50th percentile = 5614.9 g, 75th percentile = 12313.0 g, average total number of hits = 200.3; average PLA per hit = 39.5 g; Low Load cumulative PLA group: 25th percentile = 2424.6 g, 50th percentile = 2930.0 g, 75th percentile = 3800.2 g, average total number of hits = 84.1, average PLA per hit = 36.3 g). CVR measures for the High Load athletes exhibited significant whole brain and regional CVR decreases, relative to pre-season testing, that persisted at least 3–4 months after exposure ceased (Fig 6). In contrast, Low Load athletes did not experience significant CVR changes, relative to pre-season testing, at any follow-up session, in any parcellated region of interest of the brain [36]. Therefore, CVR changes in collision sport athletes are a function of cumulative loading. It follows that any controllable changes resulting in a previously designated High Load athlete shifting down to the Low Load range also corresponds to a notable decrease in risk of neurophysiological detriment.

Utilizing the data obtained here and the strong regression between the dimensionless parameters, a 19.7% reduction in PLA could result by decreasing inflation pressure from 1.10 bar (16 psi) down to 0.55 bar (8 psi), a further 7.1% reduction as a result of downsizing from a size 5, 0.55 bar (8 psi) ball to a size 4.5, 0.55 bar (8 psi) ball (Table 3), and a 20% reduction in the number of hits based on the average PLA of each impact event for each player. The High Load adjustments resulted in a 25th percentile = 2463 g, 50th percentile = 3063 g, and 75th percentile = 6718 g. This serves to demonstrate that had these game play adjustments been employed for the duration of the season, a significant portion of the High Load athletes would have dropped into the identified much safer Low Load range. Using these data to improve the design of soccer headgear could further provide additional reductions in cumulative PLA experienced by a soccer player over the entire season.

Based on the results of Svaldi et al., [36] a decrease in cumulative PLA as a result of inflation pressure reduction, ball size reduction, and overall reduction of number of hits, could allow for a substantial number of previously designated High Load athletes to drop down to the safer Low Load designation range, meaning a decreased risk of accumulating lasting changes in brain structure or function (Fig 6).

Practical implementation of a strict pressure standard, such as lowering the maximum allowable pressure, could be achieved using a hard-stop pressure regulator such that a user can set the desired pressure and the pump will cut off air input once the specified pressure has been reached.

Decreasing number of hits requires effective sensor systems to properly monitor players and careful regulation of contact practices. Decreasing number of hits could also result from a reexamination of typical game play situations promoting heading of high-speed soccer balls. Goal kicks in particular are high velocity kicks intended to reach half-field or greater distances and are often received via a header technique. Making it an infraction to head the ball in response to a goal kick could be one such addition that would limit the number of headers a player may experience in-game and would at least limit headers of particularly high velocity soccer balls.

Given wet environmental conditions, soccer balls should be cycled out of game play for new, dry soccer balls to prevent water absorption above the allowable weight gain limit according to NCAA soccer rules. After the first 15 minutes of water submersion, the size 5 soccer ball already exceeded the allowable weight gain cited in NCAA soccer rules (Table 4). Soccer ball water retention could also be addressed at the manufacturer level. Soccer ball certification standards for water resistance would ensure manufacturers are producing soccer balls that can maintain adequate water resistance for the entire game duration (90 minutes) without incurring mass absorption beyond regulation weight limits. The recommendations presented here are by no means exhaustive and are based on the preliminary findings of this study. For instance, it may be feasible to generate a similar reduction in cumulative load by decreasing the pressure in the soccer ball and enforcing strict practice and game controls on the number of head impacts allowed. Further investigative works are required to provide ample backing to initiate any substantial game play changes.

In future work, higher resolution measurement systems would lead to higher fidelity data. All kicks were performed using a typical instep kick to allow for greater control of the angle of trajectory of the soccer ball. Relying on human input for each kick added a level of variability in terms of soccer ball trajectory control. However, with such a short travel distance and a frame-by-frame approximation of velocity, variations in the trajectory angle were never extreme enough to cause significant differences in velocity measurement. With the travel distance and video capture at a frame rate of 240 fps, the velocity measurement exhibited an error of less than 4%. Examining the balance of linear momentum and balance of angular momentum of a head-neck physiology model would provide the PLA of the center of mass of the head to be compared with seasons of data collected from head impact monitoring systems. In-game collection of typical ball inflation levels would serve to validate such a model. The overarching goal is to decrease risk of incurring traumatic brain injury, particularly cumulative long term effects, and create a quantitatively definable safer and more sustainable game play environment.
